# Pyorrhea Alveolaris

**Published:** 1891-03

**Authors:** B. F. Arrington

**Affiliations:** Ashville, N. C.


THE
AMERICAN JOURNAL
—OF—
DENTAL SCIENCE.

VOL. XXIV. BALTIMORE, MARCH, 1891. NO. 11.

ARTICLE I.—ORIGINAL.

### PYORRHEA ALVEOLARIS.

BY B. F. ARRINGTON, M. D., D. D. S., ASHVILLE, N. C.

What is it and how to be treated? There is great diversity of opinion concerning the disease. Some contend that it is a constitutional disease, is hereditary, transmissible, &c., and requires to be treated with internal remedies; while others regard it a local trouble and treat it accordingly. I hold to the latter opinion and treat locally and am generally satisfied with results, and so are my patients, especially those who respect and carry out instructions as to the care of teeth and gums. There is no disease with which the human family is afflicted that has been so much discussed and written about by dentists for the past ten or twelve years as pyorrhea alveolaris, and no other disease, possibly, has been so variously treated and with as unsatisfactory results.

The dental profession are no nearer to gather to-day as to cause of the disease and best means of successful treatment for cure than when first mention of the disease was made.

Dr. W. H. Atkinson, of New York, learned and scientific, and high authority in the dental profession, has said that "pyorrhea alveolaris means pus of the alveola sockets," and Dr. C. N. Peirce, of Philadelphia, prominent and distinguished in the profession as a practitioner and teacher, says "pyorrhea is pus in the socket." Both may be correct, but I do not think so. I believe and contend that pyorrhea is pyorrhea when the sockets are perfectly healthy and free from pus, as much so as when the disease has advanced and the sockets have become involved and hold pus in quantity. The disease has a beginning and it is always at the margin of the gum around one tooth or more, frequently only one or two teeth are involved in the incipient stage of the disease, then again we will find every tooth in either or both maxillary definitely involved. The disease is easily detected and comprehended after once seen and contrasted with a gum in a normal state.

The alveola process and tooth sockets never become involved until the disease is well advanced. The name *pyorrhea alveolaris* is erroneous and leads many astray, as above stated. The alveolus is never involved or in any way affected by the disease in the beginning, therefore should not be termed *pyorrhea alveolaris* but simply pyorrhea. A disease of the gum, which in course of progress, generates pus and will, if not interfered with, sooner or later, invade and destroy the alveola structure and cause loss of teeth.

In the beginning or incipient stage of the disease there are no calcarious deposits on the teeth and never are until the disease has made definite impression upon the gums, possibly after a duration of some months. When the deposits are once formed, a cure cannot be effected until all deposits are removed from the teeth. All the constitutional treatment, mouth washes, flowing and reflowing with strong remedies the pus sockets, of which there is so much talk, packing around the teeth and deep down into the pus pockets with caustic paste, sacrificing and excising gums, application of copper bands, &c., will not effect a cure. The deposits must first be removed and effectively, or there can be no promice of relief and cure. It is often the case that we find a tooth or teeth with sanguinary or serumenal deposits well down the root and sometimes at the apex of root, which causes a pus discharge, loosening and ultimate loss of tooth or teeth, when there is no pyorrhea visible.

All is not pyorrhea alveolaris that is so called. Sanguinary deposits at or near the apex of roots is more difficult to treat than pyorrhea. The two affiictions should never be confounded. In the case of sanguinary deposit the pus is formed deep down in the socket and works up to the margin of gum, whereas in pyorrhea the pus formation commences above the margin of the alveolus and by degrees invades the entire process, to loss of same and loss of teeth. Every case of gum-waste, or recession of gum tissue we meet with is not a case of pyorrhea, far from it. In a genuine case of pyorrhea that has progressed beyond the incipient stage, we always find considerable tumefaction of gum with discharge of blood or pus, or both on application of pressure. In recession of gum (not caused by pyorrhea) we find the reverse, and I may say almost universally, instead of inflammation, tumefaction and discharge of blood and pus from the gum as in pyorrhea, the gum is often pale, contracted and shriveled. I have seen many such cases, and often recession of gum and loss of alveola process quite to the apex of the palatal roots of superior molars, with freedom from soreness or even tenderness under pressure, and no blood or pus to be found. In such cases the teeth never loosen as they do in pyorrhea. In well developed cases of pyorrhea calcarious deposits are always to be found, and blood or pus, or both, freely flow. In gum waste or recession of the gum there is no deposit as a general rule, or very rarely; nor is there discharge of blood or pus. Very unlike derangements of gum tissue. Pyorrhea, if neglected, becomes offensive and loathsome, and causes loss of teeth. Gum-waste or recession progresses slowly without any painful, disagreeable or offensive feature, and never to the extent of loss of teeth. Pyorrhea can be treated and cured as surely and effectively as other diseases, but the loss of alveolus can not be restored by any process of treatment, nor can the gum ever be reunited to the teeth as before separated by pyorrhea, but with proper treatment can be made healthy and to fit closely to the teeth and around the necks of the teeth, so that foreign matters will not be forced between the gum and teeth under ordinary usage, that much can be accomplished and nothing more. To talk about restoring alveolus to roots of teeth after having been absorbed or destroyed by disease, slow in progress, is leading astray. Whenever the alveola process has been destroyed by and through a diseased condition of the gum, or by natural process of absorption, it is gone and gone for ever, no return to be looked for. Whenever a tooth has lost by pyorrhea three fourths of its alveolus support, it should be extracted, for the remaining alveola support is not sufficient to hold the tooth firm in place during the process of masticating food. A loosened tooth in the alveolar is a local irritant and a perpetual source of annoyance and will keep the gum in an abnormal condition.

Some dentists contend that the alveolus must be scraped, chiseled or burred away before the disease can be checked. During a practice of thirty seven years, and a part of the time making a specialty of this disease, I have never met with more than two or three cases that required the removal of any portion of the process and in those cases I would have preferred to treat by direct application of pure *sulphuric acid*, (not aromatic sulphuric acid, as some advise,) but the patients lived in the country and could not return promptly for treatment. In removing deposits from the teeth, I always try to avoid touching or interfering with the alveolus as much as possible. When the deposits have been thoroughly removed and the gums properly treated, the alveolus will soon assume healthy action and perform normal function.

The alveola process in pyorrhea is never affected only as the effect of a cause, a diseased condition of the gums. This idea does not accord with the popular opinion of the day, but it is conservative and accords with reason and common sense and can be a fact nevertheless.

This is no new disease, possibly it commenced with Adam and has prevailed through all generations to the present, and is no worse now as a disease than it was hundreds of years ago, but there has never been much said about it until the past ten or fifteen years. I recollect distinctly far back in my boyhood days, fifty years or more ago, of several parties who lost many of their teeth that seemed to be perfectly sound; they would loosen gradually and drop out, or could be easily taken out with the fingers, as is the case now. Was the loss of teeth under such circumstances, that far back, caused by scurvy, as the disease was then termed, or was it pyorrhea alveolaris as it is now called? And if there is a difference, what is it and how shall it be recognized? The thing important and most important is to know how to treat and cure the malady. We may never know the cause of the disease nor be able to prevent continuation of it, nor how far back to go for first evidence of its existence, but we can treat and cure it. That is our province and duty; let us faithfully perform it to the best of our ability with the light we have to direct and guide us, and advise as best we can for the good of our patients, their comfort and health. Nothing more can be required of us. To learn how to check disease and mitigate suffering should be the zenith of our professional ambition. Some say we must go to the root of the trouble and find out the cause before we can treat successfully; very absurd. Who knows the cause of *measles* or *typhoid fever?* Yet there is treatment and cure, and so it is with pyorrhea. So far no one knows the cause, but we can treat and cure it as effectually as many other diseases, and when we have done that, we ought to be satisfied and so ought our patients. Some advise to follow up cure with after treatment. Sometimes it is requisite, but not in the majority of cases. In treating the disease I never lance or excise any portion of the gum, matters not how great the congestion may be, nor to what extent the festoons have extended. It was an old feature in practice many years ago, but never was of any benefit towards effecting a cure. When a cure is effected, give definite instructions to patients as to regular use of tooth brush, tooth pick, &c., and to keep the teeth in a cleanly condition daily, and trust to nature to perform its offices rightly and favorably as you would in any other case of disease treated and cured.

Pyorrhea alveolaris, as the disease is termed, is in my judgment a local trouble, and not constitutional or transmissible as many contend. And it is, strictly speaking, a disease of gum tissue and not of the alveolus. The alveolus becomes involved only after the disease has made considerable and definite progress, hence only secondarily effected and wastes away through the agency of unhealthy matters generated in the gums. It yields readily to local treatment, and, when cured, is cured beyond doubt or questioning, but like other diseases, there may be a return of it. "Like causes producing like effects," then treat again if called upon and repeat treatment as you would in any other abnormal state of any organ or part of the human body.

Pyorrhea is no respector of persons, old and young, (seldom below the age of eight or nine,) rich and poor, white and colored, male and female, are all liable to be troubled with the disease, some more, some less. I have treated many cases for children not more than nine years old, (but have never seen with children a case far advanced,) and onward to pass three score and ten. There is one peculiarity about the disease. It seems to produce a serious effect upon some constitutions and no unfavorable effect whatever upon others. I have known persons to be troubled with the disease for fifteen or twenty years, to the extent of loss of many teeth, and during the entire time enjoyed good health, ate heartily, slept soundly and never suffered a moment from indigestion. There are many such cases. Then I have known persons to experience great suffering, extreme physical debility, nervous prostration, dyspepsia, &c., during the continuance of the disease, and as soon as treated and cured, all distressing and unfavorable symptoms abated and in a few weeks the person would feel and look like a different being. I have in mind two gentlemen who were about equally affected with the disease (well marked typical cases) for a number of years, eighteen or twenty, both enjoyed excellent health and perfect freedom from indigestion, could eat heartily and with a relish great variety of table fare at all times without discomfort. One was a farmer by vocation and about fifty five years of age, very energetic and a laborious worker, and substited mostly upon substantial plain food in large quantity, and he used tobacco, chewed and smoked excessively. The other was a business man, lived in town, was forty-eight years of age, was rather indolent, liked to indulge leisure, was fond of high living, liked a variety of rich diet, delicacies, &c., never used tobacco, was equally healthy as the first; both were strictly temperate as to use of alcoholic drinks. One was reared and lived in a low flat, malarial country; the other reared and lived in the hill country or high-lands, free from malaria. One was light complected with delicate features, the other dark complected with coarse, strong features. Both had lost lower front teeth and several bicuspids from the effects of the disease. One had never used a tooth-brush, had sound teeth, not a decay visible. The other had used a brush occasionally until gums became diseased and sensitive, but never afterwards and had scarcely a sound tooth in his mouth Neither had any recollection of parents or any members of either family ever having suffered with such a disease and loss of teeth. Now the question arises, was the disease in either or both cases constitutional, inherited, received through transmission, or simply local, and did the same cause prevail to produce the disease in both cases? Who can decide? And what matters it, if the cause is not comprehended? Both cases were treated with the same remedy, *sulphuric acid*, and effectually cured. Multiplicity of remedies are of no avail in this disease. I have for thirty-seven years confined my practice to one line of treatment, but have at various times experimented with various remedies that have been suggested, but have never accomplished as good results as with my long tried and favorite remedy, pure sulphuric acid, diluted to suit respective cases. In the incipient stage of the disease with children or adults, the simple application of sulphuric acid and pulverized pumice with a suitable tooth-brush is all that is requisite for a cure. The disease further advanced to the formation of deposits upon the teeth requires additional treatment. The first thing to be done is to remove with appropriate, rightly shaped, smooth edge instruments (scalers) every particle of deposit; in this operation great care should be observed, for the smallest particle left adherent to the teeth will, if it comes in contact with gum tissue, prove detrimental and great hindrance to a cure. If there is neglect in this feature of the treament a cure cannot be effected; matters not how many remedies be administered, locally or otherwise. I have never yet met with a case, in which I realized necsssity for internal remedies. It is a disease which, when well advanced, will not admit of mincing treatment, but must be dealt with heroically and without fear of discomfort to patient. You must start right and push treatment forcibly regardless of a little hurt. No necessity for being rough, but persisently forcible for effect in every effort from first to last in treatment is all important and compensates, both, patient and operator. I specify the use of smooth edge instruments for removal of deposits, for I contend that deposits should never be removed with sharp edge instruments as was the old custom, and is the custom of some practitioners in this day of reform and progrees. It is, in my opinion, bad practice and unjustifiable to apply sharp edge scalers below the enamel for removal of deposits; with sharp instruments it is utterly impossible to determine definitely when all deposits are removed. If the surface of teeth under the gum is scratched or scarred with a sharp instrument, it will serve as a local irritant to the gum, and the gum cannot be restored to a normal state until the cause of trouble is removed; therefore the importance and value of smooth edge scalers. After removal of calcareous and deposits and all foreign matter from the teeth, I then brush the teeth thoroughly with a small tooth brush, using sulphuric acid and pulverized pumice. I generally shorten the brush by cutting away several rows of bristles, and cut out every other row, so that every irregularity of tooth surface may be reached and polished by the brush and pumice. The acid will find its way where needed, around the teeth and into the deepest pus pockets. The pumice serves only to cleanse and polish the crowns and necks of the teeth and as far down the roots as the bristles of the brush will reach. I vary the strength of the acid to suit respective cases, seldom use it stronger than one of acid to five of water, nor weaker than one to thirty, and in a large majority of cases do not have to repeat application. Care should always be observed to see that the acid permeates well into the pockets around the teeth. After the use of tooth brush, a camel's hair pencil brush may be used to advantage in applying the acid around the teeth. There need not be any timidity in the use of sulphuric acid on the teeth and gums if properly diluted. No harm can or will arise from use of it. Some cry out against it, but it is from ignorance and want of experience in the use of the article. A very prominent member of the profession in the city of New York proclaims for aromatic sulphuric acid, and has gone so far as to say the aromatic properties possess the merit for cure, or words to that amount. I have tried it faithfully and failed of best results, and so will any one. The pure sulphuric acid, properly diluted, is best. Experience is our best teacher. Faithfully experiment and hold fast to that which proves best is a conservative and safe line for practice.

After the application of acid I forbid the use of water for rinsing the mouth for some minutes, as I desire the acid to have full effect upon the gums and particles of deposits dislodged, and possibly some small points still adherent to the teeth. I then apply finger pressure to the gums and press firmly to the teeth, and instruct patients to do likewise several times daily, for several days, immediately after using brush, the same that I used in applying the remedy, it being better suited to the condition of teeth and gums than the majority of tooth brushes on the market. I advise the free use of the brush daily, two or three times a day, directly following treatment to keep th margins of the gums irritated and to produce or invite healthy granulation; also to prevent the accumulation of foreign matter on the teeth below the margin of the gum. For several days after treatment the gums may bleed on application of brush, but, all the better, it is desirable. I urgently request finger pressure to follow use of brush; it promotes absorption and flattens and hardens the gum. The effect must be witnessed to be rightly appreciated.

On the second or third day after removing deposits, I make a careful inspection to see if any points of deposit have been overlooked; and if any are found, I am careful to remove it. This is a very important feature in the treatment and should never be neglected. I have for some months past followed the treatment, after rinsing of mouth, with a free application of *campho-phenique* (full strength) to the gums with favorable effect. I can't say that I think it aids cure, but it seems to abate acute sensation and is unquestionably very soothing to the gums. Patients like the application and frequently ask for reapplication of it before leaving the chair.

In a large majority of cases a cure can be effected in from five to ten days if instructions are rightly carried out.

In regard to treatment for sanguinary deposits, the important and first step is to get at and remove, if possible, the deposit, all of it. In the event the deposit is so located that you can't get at it with instruments (such cases do present now and then) then remove the tooth and get rid of the trouble; it is best for the patient. I have frequently persisted in treatment for months, then had to extract. After extraction no further treatment is necessary; the gum will soon assume healthy action.

As concerns treatment for gum waste or recession of the gums, I do not feel authorized from experience to suggest treatment, for I can't say that I have ever had definite evidence of having checked progress of the disease by any line of treatment pursued. I sometimes advise the use of a mouth wash composed of pure apple cider vinegar, one part; Jamaica rum, one part; strained honey, one part; and water, four parts. To be used as follows: take about a desert or tablespoonful into the mouth once or twice daily and apply brush forcibly, especially on parts most affected. The same wash and practice is sometimes requisite, as after treatment in some cases of pyorrhea, particularly when the gums need stimulating and toning up, mostly with persons of advanced years.

It is important in the treatment of any disease to be careful not to try to do too much. Over treatment is often worse for a patient than no treatment.

A tooth brush for daily use and to be effective should be shorter and narrower than the majority of brushes on the market, and the rows of bristles should be few and far apart, not more than three or four rows crosswise the brush with space between rows, from a quarter to three eights of an inch. A compact close bristle brush is objectionable and ordinarily will do more harm than good to the gums, and in no way benefit the teeth. The object of a brush is to cleanse the teeth, every division of them above gum margin, if possible; hence the necessity for much space between rows of bristles.

In this paper I have endeavored to make a plain statement of facts in relation to my treatment of pyorrhea, pyorrhea alveolaris, Riggs' disease, scurvy, or whatever it may be termed, and kindred diseases, with a desire that others in the profession may be induced to treat the same as I have been doing for many years with good results and satisfaction to myself and patients.

The thousand and one remedies written about and proposed for treatment of pyorrhea alveolaris I have never realized any necessity for, with those I have experimented with I have found inefficient, and with them failed to accomplish best results; therefore persevere in the use of my long tried and effective remedy, *sulphuric acid*, regarding it harmless and reliable and more certain of effecting desired results than any or all other remedies.

